# A review of BRCA gene carrier demographics in Wales

**DOI:** 10.1186/bcr3783

**Published:** 2015-11-05

**Authors:** Tom Evans, Jenny Long, Georgina Devenish, Mike Lewis, Damian Bailey, Kate Gower Thomas, Alexandra Murray

**Affiliations:** 1 Cardiff and Vale University Health Board, Cardiff, UK; 2 Breast Test Wales, Cardiff, UK; 3 University of South Wales, Pontypridd, UK; 4 All Wales Genetics Service, Cardiff, UK

## Introduction

Women who inherit a mutated copy of the BRCA-1 or BRCA-2 genes have a higher lifetime risk of developing breast cancer. There have been no large epidemiological studies looking at BRCA-positive patients in the UK.

## Methods

Across the All Wales Genetics Service, individuals with confirmed BRCA mutation, since formal testing began (1995) to 1 January 2015, were included--identified from a prospectively gathered database. Genetics case notes were obtained and retrospective analysis carried out.

## Results

A total of 419 females with mean age 47 (19−81) were included in the study. Of these, 206 were identified using diagnostic testing with the remaining 213 undergoing predictive testing. Of the predictive group who subsequently had cancer, 18 (78 %) developed breast cancer. Seven (39 %) had wide local excision (WLE), six (33 %) had single mastectomy while the remaining five (28 %) had bilateral mastectomies as their primary operation. Five of the predictive group (22 %) had ovarian cancer. Of these, four (80 %) went on to have prophylactic breast surgery too. Of the 13 patients who underwent WLE or single mastectomy, four (31 %) went on to have completion risk reduction mastectomies (RRM). From the remaining 190 individuals in the predictive group with no cancer diagnosis, 102 (54 %) have had no risk reduction surgery, 32 (17 %) RRM only, 31 (16 %) BSO only and 25 (13 %) underwent both procedures.

## Conclusion

There is variation in the surgical management of BRCA positive patients in Wales. This has implications for service allocation and demands for screening for these high-risk patients.

